# Response of YPM x Ross 708 male broilers to diets containing varying inclusions of phytase, calcium butyrate, and bacitracin methylene disalicylate during the grower and finisher periods–part 2: Intestinal health and physiology

**DOI:** 10.1016/j.psj.2025.104862

**Published:** 2025-01-31

**Authors:** Joseph P. Gulizia, Zubair Khalid, Maria T. Terra-Long, Jose I. Vargas, Jose R. Hernandez, Wilmer J. Pacheco, James T. Krehling, Kenneth S. Macklin, William A. Dozier, Klint W. McCafferty, Ruediger Hauck

**Affiliations:** a Department of Poultry Science, Auburn University, Auburn, AL 36849, United States; b Department of Pathobiology, Auburn University, Auburn, AL 36849, United States; c USDA-ARS Poultry Research Unit, Mississippi State, MS 39762, United States

**Keywords:** Phytase, Calcium butyrate, Bacitracin methylene disalicylate, Broiler, Health

## Abstract

This study evaluated the effects of calcium butyrate (**CB**) and bacitracin methylene disalicylate 50 (**BMD**) combined with different phytase concentrations on broiler intestinal health and physiology. Day-old YPM x Ross 708 male broilers (2,880) were distributed in 72 floor pens and assigned to 1 of 9 treatments (8 replicates/treatment). This experiment was a factorial arrangement including 2 phytase concentrations (500 or 1,500 FTU/kg) and 4 microbiota modulating feed additive levels (**MMFA**; (1) none, (2) only CB (0.5 g/kg of diet), (3) only BMD (55 mg/kg of diet), or (4) both CB and BMD). Additionally, a negative control without phytase and MMFA was included. Intestinal permeability was assessed on d 27. Jejunum wall and cecal content samples were collected on d 28 and 42 to assess jejunum villus height (**VH**), crypt depth, tight-junction and mucin gene expression, cecal microbiome diversity, and predicted bacterial metabolic pathways. Phytase and MMFA did not influence intestinal permeability (*P* > 0.05). Combining both CB and BMD with 1,500 FTU/kg of phytase compared to 500 FTU/kg lowered d 28 VH (*P* ≤ 0.05). Jejunal expression of CL-1, CL-4, CL-5, and ZO-2 on d 28 as well as CL-2 on d 42 changed between MMFA when combined with 1,500 FTU/kg of phytase but not 500 FTU/kg (*P* ≤ 0.05). Day 42 Pielou's evenness increased when 1,500 FTU/kg of phytase was combined with both CB and BMD compared to no MMFA (*P* ≤ 0.05). The cecal microbial beta diversity was not influenced by phytase, MMFA, or their interaction (*P* > 0.05). Overall, broiler intestinal health and physiology were influenced by CB and BMD depending on phytase concentration, demonstrating the complex interactions between these feed additives.

## Introduction

The conversion of feed to muscle is the essence of broiler production. Until recently, antibacterial growth promotor (**AGP**) inclusion was an important strategy to improve production efficiency, weight gain, and feed conversion ratio (**FCR**) (Maria Cardinal et al., 2019). Bacitracin methylene disalicylate (**BMD**), a polypeptide antibiotic, has been traditionally used as an AGP to prevent diseases caused by gram-positive bacteria, such as *Clostridium perfringens* (Stutz et al., 1983). Its antimicrobial properties can help reduce pathogenic bacterial load in the intestine and modulate intestinal microbiota in a potentially positive way (Casewell et al., 2003; Crisol-Martínez et al., 2017). Due to concerns about antibacterial resistance and consumer pressure, AGP are no longer used or have limited use in most broiler-producing companies (Huyghebaert et al., 2011). However, BMD can still be used in the USA for prevention and control of necrotic enteritis.

One class of feed additives utilized to compensate for the loss of AGP are short chain fatty acids, among them butyric acid (Ayalew et al., 2022). Butyric acid or butyrate based feed additives can alter intestinal morphology (Deng et al., 2023), possess anti-inflammatory properties (Zou et al., 2019), and improve intestinal integrity (Mátis et al., 2022). They can also impact the composition and activity of the intestinal microbiota (Ficagna et al., 2022; Wan et al., 2022), such as reducing *Salmonella* colonization and shedding (Fernández-Rubio et al., 2009; Onrust et al., 2020).

Phytase, a commonly used feed additive, is an enzyme that increases phosphorus availability by liberating phosphate from phytate and reducing its anti-nutritional effects of phytate in plant-based ingredients (Selle et al., 2023). Phytase can also indirectly affect intestinal health by increasing *Lactobacillus* colonization in the jejunum and ileum (Ptak et al., 2015), altering concentrations of short chain fatty acids and villus height (Moita et al., 2021), and having an effect on the systemic immune system (Liu et al., 2008).

To optimize broiler growth, it is important to understand how phytase interacts with calcium butyrate (**CB**) and BMD in relation to enteric health and physiology measurements. This experiment investigated the individual and interactive effects of these feed additives on intestinal permeability, jejunum histomorphometry and tight-junction gene expression, as well as the diversity and metagenomic analysis of the cecal microbiome in YPM x Ross 708 male broilers.

## Materials and methods

### Animal care

This experiment was conducted at the Auburn University Charles C. Miller Jr. Poultry Research and Education Center. All procedures involving live birds were approved by the Auburn University Institutional Animal Care and Use Committee (PRN 2022-4061).

### Bird management

A total of 2,880 male d-old broiler chicks (YPM x Ross 708; Aviagen North America, Huntsville, AL) was obtained from a commercial hatchery. All chicks received a coccidiosis vaccine (Coccivac-B52, Merck Animal Health, Madison, NJ) containing live oocysts of *Eimeria* spp. (*E. acervulina, E. maxima, E. maxima MFP, E. mivati*, and *E. tenella*) at the hatchery. Upon arrival, chicks were weighed and distributed randomly among 72 floor pens (40 birds/pen, 0.07 m^2^/bird from d 1 to 27; 33 birds/pen, 0.08 m^2^/bird from d 28 to 42) in an environmentally controlled house. All floor pens contained used litter from 1 previous flock that was not subjected to a disease challenge. Each pen was top-dressed with new pine shavings. Feed and water were provided for *ad libitum* consumption.

### Feed formulation, manufacture, and experimental design

Broilers were assigned to 1 of 9 treatments with 8 replicate pens per treatment. This experiment was a 2 × 4 + 1 factorial arrangement including 2 concentrations of phytase and 4 groups of microbiota modulating feed additives (**MMFA**). Phytase was included at either 500 or 1,500 FTU/kg (Optiphos Plus, Huvepharma Inc., Peachtree City, GA). Microbiota modulating feed additives included the individual or combined supplementation of a CB product (ButiPEARL, Kemin Industries Inc., Des Moines, IA) and BMD 50 (Zoetis, Parsippany, NJ). Therefore, the 4 groups of MMFA included no additive, CB (0.5 g supplement/kg of diet), BMD (55 mg/kg of diet), or both CB and BMD. The negative control (**NC**) was devoid of feed additives. The 8 treatments containing phytase, MMFA, or both were manufactured using the NC diet composition. The phytase product had an enzyme activity of 10,000 FTU/g, as indicated on the product packaging. According to the manufacturer, the encapsulated butyrate product contained 50 % CB (0.5 g supplement/kg of diet = 0.25 g CB/kg of diet). As for BMD, 454 g of product was equivalent to 50 g of bacitracin. Ingredient and nutrient composition of experimental diets are presented in a companion manuscript with live performance measurements (Gulizia et al., 2024). Broilers were fed a 3-phase feeding program with starter feed from d 1 to 14, grower feed from d 15 to 28, and finisher feed from d 29 to 42.

### Intestinal permeability

A modified intestinal permeability protocol was followed in accordance with Baxter et al. (2017). On d 27, fluorescein isothiocyanate dextran (**FITC-d**; Sigma Aldrich Co., St. Louis, MO) was administered in a 1 mL oral gavage (8.3 mg/kg of BW) to 2 randomly selected birds per replicate. The solution was applied directly into the crop using a 1 mL-insulin syringe with a buttoned cannula attached. Birds did not undergo a fasting period before receiving the solution to avoid compromising intestinal integrity or confounding other intestinal samples (Liu et al., 2021). After 2 h, approximately 1 mL of blood was taken from the wing vein using a syringe and hypodermic needle and collected in heparin tubes (Sarstedt, Nümbrecht, Germany). Blood samples were directly placed into a cooler after collection. Samples were centrifuged (VWR International, Radnor, PA) at 1000 x g for 15 min to isolate the plasma. Afterwards, plasma samples were analyzed for fluorescence using a SpectraMax iD3 microplate reader (Molecular Devices, San Jose, CA). To determine FITC-d concentrations, a standard calibration curve was produced using sterile PBS (VWR, Solon, OH) and known concentrations of FITC-d at a 2-fold dilution series (0.00000, 0.00063, 0.00125, 0.00250, 0.00500, 0.01000, 0.02000, and 0.04000 mg/mL) in triplicates. For both the standard curve and sample plates, the plate read configuration had an excitation wavelength of 485 nm with an emission wavelength of 528 nm.

### Sampling

Five birds per replicate were randomly selected and euthanized using carbon dioxide followed by cervical dislocation on d 28 and 42. The gastrointestinal tract was removed, and the jejunum and ceca were isolated. Approximately 10 cm upstream of Meckel's diverticulum, a section of approximately 2 cm was flushed with sterile PBS (VWR, Solon, OH) and then placed into 10 % buffered formalin (VWR, Radnor, PA). From these same 5 birds, 1 g of adjacent jejunal wall was collected from 2 birds per replicate and stored for 24 h in RNAlater (Qiagen, Hilden, Germany) at 4°C. After that, RNAlater was discarded, and the sample was stored at −80°C for relative gene expression analysis. Cecal content was also collected from 2 of the 5 birds per replicate, which were randomly selected from the original 5 euthanized birds. This cecal content was collected using plastic spoons to minimize contamination with chicken DNA, immediately placed on ice, and then stored at −80°C for microbiome analysis.

### Jejunum histomorphometry

Day 28 and 42 jejunum samples were processed by the Alabama State Diagnostic Laboratory at Auburn University (Auburn, AL). Samples were embedded in paraffin, sectioned at 5 µm, mounted on glass slides, stained with hematoxylin and eosin by routine methods, and were evaluated using light microscopy. Photomicrographs were taken at 40x magnification. The height of 10 villi and the depth of 10 adjacent crypts per pen (5 birds/pen) were measured using Olympus cellSens Standard image software (version 1.12, 2014). Individual villi were randomly selected, apparently complete, and full-sized without bending or mechanical damage. Villus height (**VH**) and crypt depth (**CD**) were recorded and VH to CD ratios (**VH:CD**) were calculated. All jejunum histomorphometry measurements were averaged for each pen with the pen being the statistical unit.

### Jejunum gene expression

Ribonucleic acid was extracted from 15 mg of jejunal wall with the Qiagen RNeasy kit (Qiagen, Hilden, Germany) according to the manufacturer's instructions with DNA digestion on the column during RNA extraction with the Qiagen RNase-Free DNase Set (Qiagen, Hilden, Germany). While 2 birds per replicate were sampled, only 1 randomly selected sample per replicate plus 2 randomly selected samples (total of 10 samples/treatment) were used for analysis. Total RNA was transcribed into cDNA using the Lunascript kit (New England Biolabs, Ipswich, MA). The cDNA was diluted 1:4 in RNAse-free water. Two housekeeping genes (GAPDH and HMBS), 6 tight junction genes including claudins (CL-1, CL-2, CL-4, and CL-5) and zonula occludens (ZO-1 and ZO-2), and 1 mucus production gene (MUC-2) were amplified in duplicates by qPCR using the Fast SYBR Green Master Mix (Qiagen, Hilden, Germany). Cycling parameters consisted of an initial 10 min at 95°C denaturation cycle, 40 cycles of denaturation for 30 s at 95°C, annealing for 1 min at 60°C, and extension at 72°C for 30 s. Specificity of PCR products was confirmed by a melting curve analysis. To obtain each primer efficiency, a standard curve was calculated for each gene with a 2-fold dilution. Primers, their efficiencies, and references are listed in Table 1. Gene expression was calculated relative to the expression of 2 housekeeping genes (GAPDH and HMBS), with the average of the control birds used as a calibrator, following the method described by Vandesompele et al. (2002) and Hellemans et al. (2007).

**Table 1:** The primer sequences used for expression of targeted genes using quantitative PCR.

Target gene	Primer sequences (5′−3′)^1^	PCR efficiency	Amplicon length (base pairs)	Refs.
Claudin 1 (CL-1)	F: CTGATTGCTTCCAACCAG R: CAGGTCAAACAGAGGTACAAG	1.86	140	García et al., 2021
Claudin 2 (CL-2)	F: CCTCAGCCCTCCATCAAA R: CTGCGTCTTCTCCTCTTACTGT	2.04	162	Ozden et al., 2010
Claudin 4 (CL-4)	F: GAAGCGCTGAACCGATACCA R: TGCTTCTGTGCCTCAGTTTCC	1.85	137	Rahner et al., 2004
Claudin 5 (CL-5)	F: CATCACTTCTCCTTCGTCAGC R: GCACAAAGATCTCCCAGGTC	1.95	224	García et al., 2021
Zonula occludens 1 (ZO-1)	F: CTTCAGGTGTTTCTCTTCCTCCTC R: CTGTGGTTTCATGGCTGGATC	1.98	101	Hunziker et al., 2009
Zonula occludens 2 (ZO-2)	F: CGGCAGCTATCAGACCACTC R: CACAGACCAGCAAGCCTACAG	1.98	89	Honkatukia et al., 2011
Mucin 2 (MUC-2)	F: GCCTGCCCAGGAAATCAAG R: CGACAAGTTTGCTGGCACAT	1.97	59	Wang et al., 2019
GAPDH	F: TGGAGAAACCAGCCAAGTAT R: GCATCAAAGGTGGAGGAAT	2.07	145	Wang et al., 2018
HMBS	F: GATGGATCCGATAGCCTGAA R: GATGTGCTTAGCTCCCTTGC	2.01	195	Zhang et al., 2018

1 F = Forward; *R* = Reverse.

### Cecal microbiome

Cecal content was sampled from 2 birds per replicate. However, only 1 randomly selected sample per replicate (8 samples/treatment) was used for analysis. Deoxyribonucleic acid was extracted from 200 µL cecal content using the QIAamp DNA Stool Mini Kit (Qiagen, Hilden, Germany) following the manufacturer's instructions. Between 400 and 450 base pairs (**bp**) of the bacterial 16S rRNA gene were amplified as described by Hamilton et al. (2019) using the Taq PCR Master Mix Kit (Qiagen, Hilden, Germany). Primers 515F with linker CS1 and 926R with linker CS2 were used. Polymerase chain reaction products were verified by agarose gel electrophoresis and submitted to the University of Illinois at Chicago DNA Services Facility (Chicago, IL) for next-generation amplicon sequencing on the Illumina MiSeq. The raw reads were deposited into the NCBI Sequence Read Archive database (BioProject ID^1^: PRJNA1206084). For pre-processing of the raw reads, taxonomic classification, and analysis of diversity core metrics, a standard QIIME2 pipeline was utilized (Bolyen et al., 2019). The primers were trimmed from the reads, and sequences were denoised using a truncation length of 250 bp and a maximum expected error threshold of 4 for both forward and reverse reads using the DADA2 plugin. The taxonomic composition of the bacterial communities was assessed using the Naïve Bayes classifier trained on Silva Release 138 sourced from the SILVA ribosomal RNA gene database project (Quast et al., 2013). Sequences were filtered at a threshold of 20,000 reads for generating core diversity metrics. Cecal microbiome alpha diversity was assessed for community evenness using Pielou's evenness index (Pielou, 1966), phylogeny-based richness using Faith's phylogenetic diversity index (**FPD**; Faith, 1992), and non-phylogenetic richness termed as observed features (**OF**) in the QIIME2 program (Bolyen et al., 2019). Unweighted UniFrac distances were used to evaluate cecal microbiome beta diversity (Lozupone and Knight, 2005).

The bacterial metagenome prediction was performed using PICRUSt2 program (Douglas et al., 2020). Predicted metagenome consisted of gene names derived from Kyoto Encyclopedia of Genes and Genomes (**KEGG**) Orthology Database (Kanehisa et al., 2017). The subsequent analyses were carried out using RStudio version 2024.04.1 Build 748 (R Core Team, 2023; Posit team, 2024). The differential expression of predicted genes was computed using DESeq2 package (Love et al., 2014). Differentially expressed genes (**DEGs**) were filtered using statistical significance thresholds *P* < 0.05 and log2 fold change ≥ 1 for respective group comparisons of phytase and MMFA. Genes with a log2 fold change > 1 were considered enriched, while a log2 fold change < 1 were depleted. For pathway annotation, ClusterProfiler 4.0 (Wu et al., 2021) package was utilized. Briefly, a ranked list of DEGs was prepared based on log2 fold change values and fed into the enrichKEGG function. Top bacterial metabolic pathways were selected from relevant subcategories based on the gene ratio, and dot plots were generated using the ggplot2 package (Wickham, 2016) in RStudio version 2024.04.1 Build 748 (R Core Team, 2023; Posit team, 2024).

### Statistical analyses

This experiment was a completely randomized design with data analyzed as a 2 × 4 factorial to evaluate the main effects of phytase and MMFA and their interactions. There were 2 concentrations of phytase (500 or 1,500 FTU/kg) and 4 groups of MMFA (none, only CB, only BMD, or both CB and BMD). Excluded from this factorial analysis was the NC treatment. Instead, independent orthogonal contrasts were performed for comparison between phytase supplemented diets with no MMFA (NC + 500 FTU/kg and NC + 1,500 FTU/kg) and the NC (0 FTU/kg). In addition, orthogonal polynomial contrasts were performed for the unequally spaced phytase concentrations (0, 500, and 1,500 FTU/kg). The ORPOL function in PROC IML was used for determination of CONTRAST coefficients for the unequally spaced treatments. Independent orthogonal and orthogonal polynomial contrasts were conducted for all data except for cecal microbiome beta diversity and metagenomic analyses.

Each treatment was represented by 8 replicate pens. Pen was considered as the experimental unit for intestinal permeability, jejunum histomorphometry, cecal microbiome diversity, and metagenomic analysis, whereas bird was the experimental unit for tight-junction gene expression. Relative gene expression values were log2 transformed before statistical analysis. Using Tukey's method, outliers were determined when values were 1.5 times the interquartile range below and above the first and third quartile, respectively (Tukey, 1977). Plasma fluorescence, histomorphometry, log2 transformed relative gene expression, and cecal microbiome alpha diversity were analyzed using the GLIMMIX procedure of the SAS software version 9.4 (SAS Institute Inc., Cary, NC). Least square means were compared using the Tukey-Kramer option with statistical significance considered at *P* ≤ 0.05. For all means, 95 % confidence limits were determined. If the Tukey-Kramer test was unable to separate the means, then interactions will be described by determining the overlap of 95 % confidence limits between treatments (Cumming and Fidler, 2009).

The cecal microbiome beta diversity indices were statistically compared using UniFrac distance matrix (Lozupone and Knight, 2005) between the NC (0 FTU/kg) and NC with phytase inclusion (500 and 1,500 FTU/kg) and with a factorial design approach excluding the NC. Similarly, cecal microbiome beta diversity was compared between sampling days using the interaction treatments (Supplementary Data 1). To determine statistical significance in a multivariate analysis and generate dispersion plots, adonis function within the vegan package (Oksanen et al., 2015) was used for permutational analysis of variance in the statistical software RStudio version 2024.04.1 Build 748 (R Core Team, 2023; Posit team, 2024). Differential abundance of various genera was tested using Analysis of Compositions of Microbiomes with Bias Correction (ANCOMBC) package version 2.6.0 (Lin and Peddada, 2020) (Supplementary Data 1). The analysis included feature filtering, zero-inflation adjustment, and compositional bias correction. The output included fold changes of various taxa compared to NC group.

Analysis of differential expression of PICRUSt2-predicted genes involved a negative binomial distribution model in DESeq2 package to estimate variance-mean dependence in count data. ClusterProfiler utilizes hypergeometric tests to assess the over-representation of KEGG pathways among the DEGs and reports *P*-values following adjustment for multiple comparisons using the Benjamini-Hochberg method. A detailed account of statistics for DEGs (Supplementary Data 2) and pathways (Supplementary Data 3) were reported.

To determine the relationship between d 28 and 42 live performance measurements reported in Gulizia et al. (2024) and intestinal health and physiology measurements, Pearson correlation coefficients (Supplementary Data 1) were generated using the CORR procedure of the SAS software version 9.4 (SAS Institute Inc., Cary, NC). The analysis was performed across phytase and MMFA main effects and with the pen as the statistical unit. Strength of the correlation was determined based on the description by Pang et al. (2024): weak (|r| = 0.20-0.39), moderate (|r| = 0.40-0.59), strong (|r| = 0.60-0.79), and very strong (|r| = 0.80-0.99). The performance measurements considered for analysis were BW, FCR, CP digestibility, and apparent ileal digestible energy (**AIDE**), whereas the intestinal health and physiology measurements were VH, VH:CD ratio, Pielou's evenness index, and OF.

## Results

### Intestinal permeability

Independent orthogonal and orthogonal polynomial contrasts between the NC (0 FTU/kg) and NC with phytase inclusion (500 and 1,500 FTU/kg) on intestinal permeability were not different (*P* > 0.05) (Table 2). Broiler intestinal permeability on d 27 was not influenced by phytase or MMFA (*P* > 0.05) (Table 3).

**Table 2:** Independent orthogonal and orthogonal polynomial contrasts^1^ for intestinal permeability and jejunal villus height (VH), crypt depth (CD), and their ratio (VH:CD) between YPM x Ross 708 male broilers provided a negative control (NC; 0 FTU/kg) diet and a NC with phytase supplementation (500 or 1,500 FTU/kg) from 1 to 42 d of age^2^.

	d 27	d 28			d 42		
Item	FITC-d^3^, mg/mL	VH, µm	CD, µm	VH:CD ratio	VH, µm	CD, µm	VH:CD ratio
NC vs. NC + phytase^4^							
NC	0.0047	966	204	4.86	1,101	192	5.84
NC + 500 FTU/kg	0.0047	973	201	5.01	1,085	170	6.56
NC + 1,500 FTU/kg	0.0047	986	212	4.79	1,071	170	6.51
CLM^5^	± 0.0006	± 58	± 15	± 0.35	± 66	± 11	± 0.47
	*P-value*						
NC vs. NC + 500 FTU/kg	0.968	0.866	0.809	0.522	0.731	0.008	0.032
NC vs. NC + 1,500 FTU/kg	0.957	0.638	0.436	0.799	0.525	0.008	0.047
Linear	0.960	0.633	0.367	0.677	0.536	0.018	0.089
Quadratic	0.981	0.990	0.569	0.412	0.882	0.045	0.091

1 Orthogonal polynomial contrasts were performed for the unequally spaced phytase concentrations (0, 500, and 1,500 FTU/kg).

2 Intestinal permeability: values are least square means of 8 replicate pens, with each pen having blood collected from 2 broilers on d 27; Jejunum histomorphometry: values are least square means of 8 replicate pens, with each pen having jejunum samples collected from 5 broilers (duplicated; *n* = 10/pen) on d 28 and 42; statistical significance was considered at *P* ≤ 0.05.

3 Intestinal permeability was assessed by determining the amount of fluorescein isothiocyanate dextran (FITC-d) concentration in the blood.

4 OptiPhos Plus (Huvepharma Inc., Peachtree City, GA) provided 500 FTU/kg or 1,500 FTU/kg of phytase activity per kg of diet.

5 CLM = 95 % confidence limit for the mean.

**Table 3:** Intestinal permeability and jejunal villus height (VH), crypt depth (CD), and their ratio (VH:CD) of YPM x Ross 708 male broilers provided a negative control diet varying in phytase, calcium butyrate (CB), and bacitracin methylene disalicylate (BMD) inclusion from 1 to 42 d of age^1^.

			d 27	d 28			d 42		
Item			FITC-d^2^, mg/mL	VH, µm	CD, µm	VH:CD ratio	VH, µm	CD, µm	VH:CD ratio
Phytase^3^, FTU/kg, main effect (*n* = 32)									
500			0.0048	1,013	207	5.03	1,074	167	6.60
1,500			0.0049	991	199	5.11	1,074	167	6.57
CLM^4^			± 0.0002	± 30	± 7	± 0.17	± 32	± 6	± 0.24
Microbiota modulating feed additives, main effect (*n* = 16)									
None			0.0047	979	207	4.90	1,078^ab^	170	6.53
Only CB^5^			0.0050	1,011	207	4.99	1,142^a^	170	6.85
Only BMD^6^			0.0048	1,002	199	5.17	1,042^b^	171	6.27
Both CB and BMD			0.0050	1,016	199	5.21	1,033^b^	158	6.70
CLM			± 0.0003	± 43	± 10	± 0.24	± 45	± 8	± 0.34
Phytase, FTU/kg	x	Microbiota modulating feed additives							
500		None	0.0047	973^ab^	201	5.01	1,085	170	6.56
500		Only CB	0.0049	995^ab^	217	4.67	1,132	170	6.80
500		Only BMD	0.0047	1,001^ab^	202	5.08	1,062	174	6.30
500		Both CB and BMD	0.0051	1,083^a^	207	5.36	1,016	154	6.76
1,500		None	0.0047	986^ab^	212	4.79	1,071	170	6.51
1,500		Only CB	0.0051	1,028^ab^	197	5.31	1,153	170	6.90
1,500		Only BMD	0.0050	1,003^ab^	197	5.27	1,021	169	6.24
1,500		Both CB and BMD	0.0050	948^b^	191	5.07	1,049	161	6.65
CLM			± 0.0005	± 60	± 15	± 0.34	± 63	± 11	± 0.48
			*P-value*						
Phytase			0.457	0.305	0.157	0.512	0.999	0.943	0.867
Microbiota modulating feed additives			0.453	0.633	0.552	0.224	0.004	0.070	0.106
Phytase x Microbiota modulating feed additives			0.611	0.030	0.184	0.029†	0.627	0.728	0.975

^a,b^Means within a column with different superscripts differ significantly (*P* ≤ 0.05).

1 Intestinal permeability: interaction values are least square means of 8 replicate pens, with each pen having blood collected from 2 broilers on d 27; Jejunum histomorphometry: interaction values are least square means of 8 replicate pens, with each pen having jejunum samples collected from 5 broilers (duplicated; *n* = 10/pen) on d 28 and 42.

2 Intestinal permeability was assessed by determining the amount of fluorescein isothiocyanate dextran (FITC-d) concentration in the blood.

3 OptiPhos Plus (Huvepharma Inc., Peachtree City, GA) provided 500 FTU/kg or 1,500 FTU/kg of phytase activity per kg of diet.

4 CLM = 95 % confidence limit for the mean.

5 ButiPEARL (Kemin Industries Inc., Des Moines, IA) included at 0 or 0.5 g supplement/kg of diet.

6 Bacitracin methylene disalicylate 50 Type A Medicated Article (Zoetis, Parsippany, NJ) included at 0 or 55 mg per kg of diet.

† Overall interaction was significant; however, Tukey-Kramer multiple comparison test did not separate the means.

### Jejunum histomorphometry

Independent orthogonal and orthogonal polynomial contrasts between the NC (0 FTU/kg) and NC with phytase inclusion (500 and 1500 FTU/kg) on d 28 histomorphometry were not different (*P* > 0.05) (Table 2). However, there were differences between the NC and NC with phytase inclusion on d 42 histomorphometry as indicated by the independent orthogonal and orthogonal polynomial contrasts (Table 2). Broilers consuming the NC devoid of phytase had an increased d 42 CD and lowered VH:CD ratio compared to both phytase supplemented groups (*P* ≤ 0.05). A quadratic response to phytase supplementation was observed for d 42 CD (*P* ≤ 0.05).

Day 28 and 42 jejunum VH, CD, and VH:CD ratios are shown in Table 3. Interactions between phytase and MMFA were observed for d 28 VH and VH:CD ratio (*P* ≤ 0.05). Within each phytase group, d 28 VH was similar between all levels of MMFA. However, d 28 VH increased when both CB and BMD were combined with 500 FTU/kg of phytase compared to 1500 FTU/kg (*P* ≤ 0.05). At a phytase concentration of 1500 FTU/kg, d 28 VH:CD ratio across treatments showed minimal variation. However, with 500 FTU/kg of phytase, including both CB and BMD in the diet increased d 28 VH:CD ratio compared to only CB (*P* ≤ 0.05). Phytase and MMFA did not influence d 28 CD (*P* > 0.05). Irrespective of MMFA, d 42 jejunum histomorphometry was not different between phytase concentrations (*P* > 0.05). Only d 42 VH was influenced by MMFA. Including only CB in the diet increased VH compared to including either only BMD or both CB and BMD (*P* ≤ 0.05).

### Jejunum gene expression

Only d 28 CL-1 and CL-2 expressions were different between the NC (0 FTU/kg) and NC with phytase inclusion (500 and 1,500 FTU/kg) as indicated by the independent orthogonal and orthogonal polynomial contrasts (Table 4). Claudin-1 was downregulated more when broilers consumed NC + 1,500 FTU/kg compared to the NC with no phytase (*P* ≤ 0.05). A linear response to increasing phytase concentrations was observed for CL-1 expression (*P* ≤ 0.05). More pronounced downregulation of CL-1 expression occurred as phytase concentrations increased. Additionally, downregulation of CL-2 occurred when broilers consumed the NC + 500 FTU/kg compared to NC (*P* ≤ 0.05).

**Table 4:** Independent orthogonal and orthogonal polynomial contrasts^1^ for tight junction (CL-1, CL-2, CL-4, CL-5, ZO-1, and ZO-2) and mucin (MUC-2) gene expression between YPM x Ross 708 male broilers provided a negative control (NC; 0 FTU/kg) diet and a NC with phytase supplementation (500 or 1500 FTU/kg) on d 28^2^.

		Gene expression, Log2						
Item		CL-1	CL-2	CL-4	CL-5	ZO-1	ZO-2	MUC-2
NC vs. NC + phytase^3^								
NC		−0.278	0.171	−1.387	−0.095	0.083	0.085	0.357
NC + 500 FTU/kg		−0.436	−0.650	−0.990	−0.043	0.297	0.071	0.825
NC + 1,500 FTU/kg		−1.004	−0.340	−1.330	−0.507	0.010	0.048	1.052
CLM^4^		± 0.392	± 0.561	± 1.128	± 0.434	± 0.291	± 0.349	± 0.774
		*P-value*						
NC vs. NC + 500 FTU/kg		0.563	0.043	0.612	0.867	0.303	0.954	0.373
NC vs. NC + 1,500 FTU/kg		0.008	0.204	0.941	0.185	0.727	0.881	0.199
Linear		0.005	0.345	0.976	0.139	0.545	0.880	0.227
Quadratic		0.728	0.068	0.580	0.489	0.195	0.993	0.611

1 Orthogonal polynomial contrasts were performed for the unequally spaced phytase concentrations (0, 500, and 1,500 FTU/kg).

2 Values are least square means of 10 broilers per treatment, with each pen having jejunum samples collected on d 28; statistical significance was considered at *P* ≤ 0.05.

3 OptiPhos Plus (Huvepharma Inc., Peachtree City, GA) provided 500 FTU/kg or 1,500 FTU/kg of phytase activity per kg of diet.

4 CLM = 95 % confidence limit for the mean.

Dietary inclusion of phytase and MMFA influenced d 28 tight junction gene expression (Table 5). Compared to no inclusion of MMFA, CL-2 expression was upregulated with dietary inclusion of only BMD (*P* ≤ 0.05). Phytase and MMFA interactions were observed for CL-1, CL-4, CL-5, and ZO-2 expression (*P* ≤ 0.05). Within the 500 FTU/kg group, expressions of CL-1, CL-4, CL-5, and ZO-2 were not influenced by CB, BMD, and their combination. Therefore, MMFA responded differently with a dietary inclusion of 1,500 FTU/kg of phytase. Claudin-1 expression was highest with the inclusion of only CB. Additionally, CL-1 expression increased with only BMD compared to no MMFA (*P* ≤ 0.05). Supplementation of only CB increased CL-4 and ZO-2 expression compared to the other MMFA groups (*P* ≤ 0.05). Claudin-5 expression was higher when broilers consumed a diet with only CB compared to no MMFA (*P* ≤ 0.05).

**Table 5:** Tight junction (CL-1, CL-2, CL-4, CL-5, ZO-1, and ZO-2) and mucin (MUC-2) gene expression of YPM x Ross 708 male broilers provided a negative control diet varying in phytase, calcium butyrate (CB), and bacitracin methylene disalicylate (BMD) inclusion on d 28^1^.

			Gene expression, Log2						
Item			CL-1	CL-2	CL-4	CL-5	ZO-1	ZO-2	MUC-2
Phytase^2^, FTU/kg, main effect (*n* = 40)									
500			−0.708	0.059	−1.456	−0.242	0.188	0.012	0.739
1,500			0.020	−0.229	1.346	0.075	0.338	0.766	0.806
CLM^3^			± 0.197	± 0.269	± 0.568	± 0.228	± 0.159	± 0.175	± 0.364
Microbiota modulating feed additives, main effect (*n* = 20)									
None			−0.720	−0.495^b^	−1.160	−0.275	0.154	0.059	0.938
Only CB^4^			0.431	−0.081^ab^	2.237	−0.008	0.094	1.203	0.918
Only BMD^5^			−0.435	0.228^a^	−1.218	−0.147	0.435	0.156	0.734
Both CB and BMD			−0.652	−0.052^ab^	−0.078	0.094	0.370	0.138	0.500
CLM			± 0.286	± 0.391	± 0.850	± 0.327	± 0.232	± 0.270	± 0.529
Phytase, FTU/kg	x	Microbiota modulating feed additives							
500		None	−0.436^bc^	−0.650	−0.990^b^	−0.043^ab^	0.297	0.071^b^	0.825
500		Only CB	−1.007^c^	0.310	−2.140^b^	−0.545^b^	−0.039	0.077^b^	1.410
500		Only BMD	−0.791^bc^	0.313	−2.243^b^	−0.336^ab^	0.169	−0.125^b^	0.251
500		Both CB and BMD	−0.598^bc^	0.264	−0.450^b^	−0.045^ab^	0.326	0.026^b^	0.471
1,500		None	−1.004^c^	−0.340	−1.330^b^	−0.507^b^	0.010	0.048^b^	1.052
1,500		Only CB	1.869^a^	−0.473	6.614^a^	0.529^a^	0.226	2.329^a^	0.426
1,500		Only BMD	−0.078^b^	0.263	−0.192^b^	0.042^ab^	0.701	0.436^b^	1.218
1,500		Both CB and BMD	−0.706^bc^	−0.367	0.294^b^	0.234^ab^	0.414	0.249^b^	0.529
CLM			± 0.429	± 0.569	± 1.275	± 0.488	± 0.337	± 0.395	± 0.770
			*P-value*						
Phytase			<0.001	0.127	<0.001	0.051	0.187	<0.001	0.792
Microbiota modulating feed additives			<0.001	0.033	<0.001	0.366	0.101	<0.001	0.587
Phytase x Microbiota modulating feed additives			<0.001	0.144	<0.001†	0.012	0.066	<0.001	0.069

^a-c^Means within a column with different superscripts differ significantly (*P* ≤ 0.05).

1 Interaction values are least square means of 10 broilers per treatment, with each pen having jejunum samples collected on d 28.

2 OptiPhos Plus (Huvepharma Inc., Peachtree City, GA) provided 500 FTU/kg or 1,500 FTU/kg of phytase activity per kg of diet.

3 CLM = 95 % confidence limit for the mean.

4 ButiPEARL (Kemin Industries Inc., Des Moines, IA) included at 0 or 0.5 g supplement/kg of diet.

5 Bacitracin methylene disalicylate 50 Type A Medicated Article (Zoetis, Parsippany, NJ) included at 0 or 55 mg per kg of diet.

† Conservative Tukey-Kramer grouping for interaction.

There were multiple differences between the NC (0 FTU/kg) and NC with phytase inclusion (500 and 1,500 FTU/kg) on d 42 tight junction and mucin gene expressions as indicated by the independent orthogonal and orthogonal polynomial contrasts (Table 6). Day 42 CL-2 expression was more downregulated when broilers consumed the NC diet with 1,500 FTU/kg compared to 0 FTU/kg (*P* ≤ 0.05). A linear response to increasing phytase concentrations was observed for CL-2 expression (*P* ≤ 0.05). As phytase concentrations increased, a more pronounced downregulation of CL-2 expression occurred. Compared to a diet devoid of phytase, ZO-2 expression was downregulated with dietary inclusion of either 500 or 1500 FTU/kg (*P* ≤ 0.05; Linear: *P* ≤ 0.05). Lastly, dietary inclusion of 1500 FTU/kg of phytase downregulated MUC-2 compared to NC (*P* ≤ 0.05). Day 42 expression of MUC-2 linearly decreased with higher concentrations of phytase (*P* ≤ 0.05).

**Table 6:** Independent orthogonal and orthogonal polynomial contrasts^1^ for tight junction (CL-1, CL-2, CL-4, CL-5, ZO-1, and ZO-2) and mucin (MUC-2) gene expression between YPM x Ross 708 male broilers provided a negative control (NC; 0 FTU/kg) diet and a NC with phytase supplementation (500 or 1500 FTU/kg) on d 42^2^.

		Gene expression, Log2						
Item		CL-1	CL-2	CL-4	CL-5	ZO-1	ZO-2	MUC-2
NC vs. NC + phytase^3^								
NC		−0.295	−0.183	−1.216	0.004	−0.110	0.123	0.391
NC + 500 FTU/kg		−0.395	−0.517	−0.714	−0.093	−0.071	−0.335	0.292
NC + 1,500 FTU/kg		−0.507	−1.943	−2.331	0.090	−0.161	−0.762	−1.123
CLM^4^		± 0.269	± 0.805	± 1.597	± 0.405	± 0.284	± 0.292	± 0.650
		*P-value*						
NC vs. NC + 500 FTU/kg		0.602	0.527	0.642	0.735	0.847	0.022	0.823
NC vs. NC + 1,500 FTU/kg		0.258	0.002	0.315	0.767	0.797	<0.001	<0.001
Linear		0.261	0.001	0.229	0.692	0.745	<0.001	<0.001
Quadratic		0.862	0.585	0.350	0.620	0.753	0.355	0.285

1 Orthogonal polynomial contrasts were performed for the unequally spaced phytase concentrations (0, 500, and 1,500 FTU/kg).

2 Values are least square means of 10 broilers per treatment, with each pen having jejunum samples collected on d 42; statistical significance was considered at *P* ≤ 0.05.

3 OptiPhos Plus (Huvepharma Inc., Peachtree City, GA) provided 500 FTU/kg or 1,500 FTU/kg of phytase activity per kg of diet.

4 CLM = 95 % confidence limit for the mean.

Day 42 MUC-2 expression was upregulated with 500 FTU/kg compared to 1500 FTU/kg (Table 7) (*P* ≤ 0.05). Irrespective of phytase, inclusion of both CB and BMD upregulated CL-1 while including single or combinations of MMFA reduced the degree of downregulation for ZO-2 (*P* ≤ 0.05). Expression of MUC-2 was upregulated with dietary supplementation of only CB compared to no MMFA (*P* ≤ 0.05). A phytase and MMFA interaction was only observed for d 42 CL-2 expression (*P* ≤ 0.05). Expression of CL-2 was not influenced by MMFA in the 500 FTU/kg group. However, within the 1,500 FTU/kg group, the degree of downregulation was reduced with only CB compared to no MMFA (*P* ≤ 0.05).

**Table 7:** Tight junction (CL-1, CL-2, CL-4, CL-5, ZO-1, and ZO-2) and mucin (MUC-2) gene expression of YPM x Ross 708 male broilers provided a negative control diet varying in phytase, calcium butyrate (CB), and bacitracin methylene disalicylate (BMD) inclusion on d 42^1^.

			Gene expression, Log2						
Item			CL-1	CL-2	CL-4	CL-5	ZO-1	ZO-2	MUC-2
Phytase^2^, FTU/kg, main effect (*n* = 40)									
500			−0.248	−0.111	−0.711	0.026	0.029	−0.258	0.134^a^
1,500			−0.365	−0.899	−0.191	0.181	−0.002	−0.275	−0.430^b^
CLM^3^			± 0.134	± 0.368	± 0.775	± 0.220	± 0.144	± 0.133	± 0.317
Microbiota modulating feed additives, main effect (*n* = 20)									
None			−0.451^b^	−1.230	−1.522	−0.002	−0.116	−0.548^b^	−0.415^b^
Only CB^4^			−0.385^b^	−0.012	−0.642	−0.002	0.042	−0.163^a^	0.449^a^
Only BMD^5^			−0.393^b^	−0.255	−0.053	0.063	−0.109	−0.180^a^	−0.362^ab^
Both CB and BMD			0.003^a^	−0.522	0.414	0.353	0.236	−0.175^a^	−0.265^ab^
CLM			± 0.192	± 0.536	± 1.082	± 0.321	± 0.206	± 0.190	± 0.456
Phytase, FTU/kg	x	Microbiota modulating feed additives							
500		None	−0.395	−0.517^abc^	−0.714	−0.093	−0.071	−0.335	0.292
500		Only CB	−0.323	0.065^ab^	−0.853	0.149	0.099	−0.212	0.854
500		Only BMD	−0.206	0.590^a^	−0.701	−0.351	−0.280	−0.275	−0.390
500		Both CB and BMD	−0.067	−0.580^abc^	−0.576	0.398	0.366	−0.211	−0.223
1,500		None	−0.507	−1.943^c^	−2.331	0.090	−0.161	−0.762	−1.123
1,500		Only CB	−0.447	−0.090^ab^	−0.432	−0.152	−0.016	−0.115	0.044
1,500		Only BMD	−0.580	−1.100^bc^	0.595	0.476	0.062	−0.086	−0.334
1,500		Both CB and BMD	0.073	−0.464^abc^	1.404	0.309	0.105	−0.138	−0.307
CLM			± 0.271	± 0.799	± 1.570	± 0.493	± 0.291	± 0.292	± 0.680
			*P-value*						
Phytase			0.217	0.004	0.341	0.308	0.759	0.855	0.013
Microbiota modulating feed additives			0.005	0.012	0.078	0.293	0.051	0.011	0.028
Phytase x Microbiota modulating feed additives			0.309	0.035	0.112	0.056	0.169	0.096	0.068

^a-c^Means within a column with different superscripts differ significantly (*P* ≤ 0.05).

1 Interaction values are least square means of 10 broilers per treatment, with each pen having jejunum samples collected on d 42.

2 OptiPhos Plus (Huvepharma Inc., Peachtree City, GA) provided 500 FTU/kg or 1,500 FTU/kg of phytase activity per kg of diet.

3 CLM = 95 % confidence limit for the mean.

4 ButiPEARL (Kemin Industries Inc., Des Moines, IA) included at 0 or 0.5 g supplement/kg of diet.

5 Bacitracin methylene disalicylate 50 Type A Medicated Article (Zoetis, Parsippany, NJ) included at 0 or 55 mg per kg of diet.

### Cecal microbiome

**Fig. 1:**
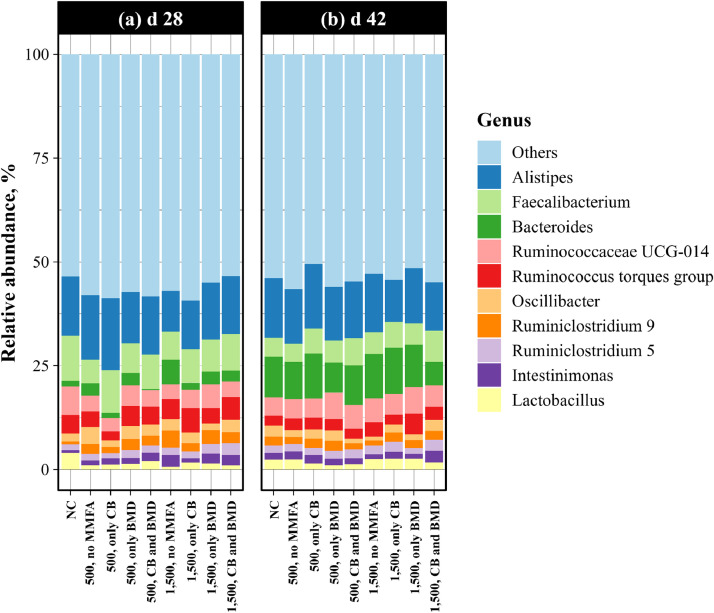
Relative abundance of the 10 most abundant genera in the cecal microbiome of YPM x Ross 708 male broilers provided a negative control (NC) diet varying in phytase (500 or 1500 FTU/kg) and microbiota modulating feed additive inclusion (MMFA; none, only calcium butyrate (CB), only bacitracin methylene disalicylate (BMD), or both CB and BMD) on (**a**) d 28 and (**b**) d 42.

Relative abundance of the 10 most abundant genera in the cecal microbiome on d 28 and 42 are shown in Fig. 1 (**a**) and (**b**), respectively. Excluding the others, *Alistipes, Faecalibacterium*, and *Ruminococcaceae UCG-014* were the most common genera found in the cecal microbiome on d 28, whereas *Alistipes, Bacteroides*, and *Faecalibacterium* were the most abundant on d 42. For both d 28 and 42, there were no apparent differences between the treatments. Additional details on the log fold change in bacterial genera are provided in Supplementary Data 1 (Fig. S1).

Independent orthogonal and orthogonal polynomial contrasts between the NC and NC with phytase inclusion were not different for d 28 and 42 cecal microbiome alpha diversity characteristics (*P* > 0.05) (Table 8), except for d 28 Pielou's evenness, which quadratically responded to phytase concentrations (*P* ≤ 0.05).

**Table 8:** Independent orthogonal and orthogonal polynomial contrasts^1^ for cecal microbiome alpha diversity characteristics between YPM x Ross 708 male broilers provided a negative control (NC; 0 FTU/kg) diet and a NC with phytase supplementation (500 or 1500 FTU/kg) on d 28 and 42^2^.

	d 28			d 42		
Item	Evenness^3^	FPD^3^	OF^3^	Evenness	FPD	OF
NC vs. NC + phytase^4^						
NC	0.785	14.52	322	0.766	17.36	441
NC + 500 FTU/kg	0.760	14.33	330	0.778	18.20	461
NC + 1,500 FTU/kg	0.799	14.91	360	0.747	17.25	398
CLM^5^	± 0.020	± 0.89	± 32	± 0.023	± 1.15	± 43
	*P-value*					
NC vs. NC + 500 FTU/kg	0.072	0.734	0.682	0.430	0.275	0.464
NC vs. NC + 1,500 FTU/kg	0.326	0.504	0.080	0.196	0.888	0.145
Linear	0.144	0.432	0.070	0.109	0.689	0.079
Quadratic	0.017	0.518	0.820	0.155	0.184	0.153

1 Orthogonal polynomial contrasts were performed for the unequally spaced phytase concentrations (0, 500, and 1,500 FTU/kg).

2 Values are least square means of 8 replicate pens, with each pen having cecal samples collected on d 28 and 42; statistical significance was considered at *P* ≤ 0.05.

3 Evenness = Pielou's evenness; FPD = Faith's phylogenetic diversity; OF = Observed features.

4 OptiPhos Plus (Huvepharma Inc., Peachtree City, GA) provided 500 FTU/kg or 1,500 FTU/kg of phytase activity per kg of diet.

5 CLM = 95 % confidence limit for the mean.

Day 28 and 42 cecal microbiome alpha diversity characteristics are shown in Table 9. A higher d 28 OF was observed with dietary inclusion of 1,500 FTU/kg of phytase compared to 500 FTU/kg (*P* ≤ 0.05). An interaction between phytase and MMFA was only observed for Pielou's evenness on d 28 (*P* ≤ 0.05). Within the 500 FTU/kg group, evenness was increased with inclusion of only BMD compared to only CB and no MMFA (*P* ≤ 0.05). In contrast, evenness was similar between all MMFA within the 1,500 FTU/kg group. Phytase, MMFA, and their combination did not influence d 42 FPD (*P* > 0.05). However, interactions were observed for d 42 Pielou's evenness and OF (*P* ≤ 0.05). Microbiota modulating feed additives did not influence evenness within the 500 FTU/kg group. However, within the 1500 FTU/kg group, d 42 evenness increased when supplementing both CB and BMD compared to no MMFA (*P* ≤ 0.05). Although an interaction was observed for d 42 OF, the Tukey-Kramer multiple comparison test did not separate the means, and the 95 % confidence intervals overlapped across all treatments.

**Table 9:** Cecal microbiome alpha diversity characteristics of YPM x Ross 708 male broilers provided a negative control diet varying in phytase, calcium butyrate (CB), and bacitracin methylene disalicylate (BMD) inclusion on d 28 and 42^1^.

			d 28			d 42		
Item			Evenness^2^	FPD^2^	OF^2^	Evenness	FPD	OF
Phytase^3^, FTU/kg, main effect (*n* = 40)								
500			0.779	15.00	349^b^	0.765	17.28	426
1,500			0.795	15.41	375^a^	0.771	17.47	427
CLM^4^			± 0.011	± 0.41	± 16	± 0.010	± 0.52	± 20
Microbiota modulating feed additives, main effect (*n* = 20)								
None			0.779	14.62	345	0.763	17.72	429
Only CB^5^			0.772	15.49	360	0.767	17.22	422
Only BMD^6^			0.796	15.37	370	0.769	17.71	443
Both CB and BMD			0.800	15.34	372	0.772	16.85	412
CLM			± 0.017	± 0.58	± 23	± 0.015	± 0.75	± 28
Phytase, FTU/kg	x	Microbiota modulating feed additives						
500		None	0.760^bc^	14.33	330	0.778^ab^	18.20	461
500		Only CB	0.747^c^	15.22	341	0.761^ab^	16.73	413
500		Only BMD	0.815^a^	15.57	373	0.776^ab^	17.56	443
500		Both CB and BMD	0.795^abc^	14.87	352	0.743^b^	16.63	388
1,500		None	0.799^ab^	14.91	360	0.747^b^	17.25	398
1,500		Only CB	0.797^abc^	15.76	379	0.772^ab^	17.70	430
1,500		Only BMD	0.777^abc^	15.17	366	0.762^ab^	17.86	443
1,500		Both CB and BMD	0.806^ab^	15.81	393	0.802^a^	17.08	436
CLM			± 0.024	± 0.88	± 33	± 0.022	± 1.06	± 43
			*P-value*					
Phytase			0.051	0.146	0.026	0.401	0.598	0.973
Microbiota modulating feed additives			0.042	0.149	0.310	0.829	0.278	0.440
Phytase x Microbiota modulating feed additives			<0.001	0.365	0.417	<0.001	0.285	0.040†

^a-c^Means within a column with different superscripts differ significantly (*P* ≤ 0.05).

1 Interaction values are least square means of 8 replicate pens, with each pen having cecal samples collected on d 28 and 42.

2 Evenness = Pielou's evenness; FPD = Faith's phylogenetic diversity; OF = Observed features.

3 OptiPhos Plus (Huvepharma Inc., Peachtree City, GA) provided 500 FTU/kg or 1,500 FTU/kg of phytase activity per kg of diet.

4 CLM = 95 % confidence limit for the mean.

5 ButiPEARL (Kemin Industries Inc., Des Moines, IA) included at 0 or 0.5 g supplement/kg of diet.

6 Bacitracin methylene disalicylate 50 Type A Medicated Article (Zoetis, Parsippany, NJ) included at 0 or 55 mg per kg of diet.

† Overall interaction was significant; however, Tukey-Kramer multiple comparison test did not separate the means.

Day 28 (*P* = 0.470) and 42 (*P* = 0.148) cecal microbiome beta diversity was not different between the NC (0 FTU/kg) and NC with 500 or 1,500 FTU/kg of phytase (data not shown). Phytase (*P* = 0.371), MMFA (*P* = 0.196), and their interaction (*P* = 0.175; Supplementary Data 1, Fig. S2) did not influence d 28 cecal microbiome beta diversity (main effect data not shown). Similarly for d 42 cecal microbiome beta diversity, phytase (*P* = 0.695), MMFA (*P* = 0.642), and their interaction (*P* = 0.070; Supplementary Data 1, Fig. S3) were not different (main effect data not shown).

Number of up- and down-regulated DEGs based on predicted metagenome of the cecal microbiota compared between the NC (0 FTU/kg) and NC with phytase inclusion (500 and 1500 FTU/kg) is shown in Table 10, while the influence of phytase and MMFA on DEGs is presented in Table 11. Supplementing phytase at 500 FTU/kg (d 28: 103 DEGs compared to the NC; d 42: 1340 DEGs compared to the NC) and 1500 FTU/kg (d 28: 184 DEGs compared to the NC) had the most influence on the inferred metabolic activity of the microbiota. However, the effect was inconsistent, with the lower dose of phytase having a much higher impact on d 42 and the higher dose showing a moderate impact on d 28. Furthermore, there were 2 common DEGs between d 28 and 42 of the 1443 genes influenced by 500 FTU/kg of phytase. These DEGs were involved in small acid-soluble spore protein I (K06426) and two-component system, OmpR family, sensor histidine kinase ArlS (K18940). Between d 28 and 42, only 58 unique DEGs were observed from the dietary inclusion of CB, BMD, or both feed additives compared to no MMFA.

**Table 10:** Number of up- and down-regulated differentially expressed genes (DEGs) based on predicted metagenome of the cecal microbiota compared between YPM x Ross 708 male broilers provided a negative control (NC; 0 FTU/kg) diet and a NC with phytase^1^ supplementation (500 or 1500 FTU/kg) on d 28 and 42.

Comparison	d 28		d 42		Unique DEGs	Intersections^2^
	Up	Down	Up	Down		
NC + 500 FTU/kg vs. NC	16	87	7	1,333	1,439	2
NC + 1,500 FTU/kg vs. NC	100	84	0	4	188	0

1 OptiPhos Plus (Huvepharma Inc., Peachtree City, GA).

2 Intersections indicate the common DEGs observed on both d 28 and 42.

**Table 11:** Number of up- and down-regulated differentially expressed genes (DEGs) based on predicted metagenome of the cecal microbiota in YPM x Ross 708 male broilers provided a negative control diet varying in phytase^1^, calcium butyrate^2^ (CB), and bacitracin methylene disalicylate^3^ (BMD) inclusion on d 28 and 42.

Comparison	d 28		d 42		Unique DEGs	Intersections^4^
	Up	Down	Up	Down		
Phytase^5^						
1,500 vs. 500 FTU/kg	0	4	0	0	4	0
MMFA^6^						
All other MMFA^7^ vs. None	3	0	0	0	3	0
Only CB vs. None	0	0	0	0	0	0
Only BMD vs. None	1	6	0	1	8	0
Both CB and BMD vs. None	0	4	35	8	47	0

1 OptiPhos Plus (Huvepharma Inc., Peachtree City, GA).

2 ButiPEARL (Kemin Industries Inc., Des Moines, IA) included at 0 or 0.5 g supplement/kg of diet.

3 BMD 50 Type A Medicated Article (Zoetis, Parsippany, NJ) included at 0 or 55 mg per kg of diet.

4 Intersections indicate the common DEGs observed on both d 28 and 42.

5 Phytase (FTU/kg) includes: (1) 500 and (2) 1,500.

6 Microbiota modulating feed additives includes: (1) none, (2), only CB, (3) only BMD, and (4) both CB and BMD.

7 All other MMFA includes: (2) only CB, (3) only BMD, and (4) both CB and BMD.

**Fig. 2:**
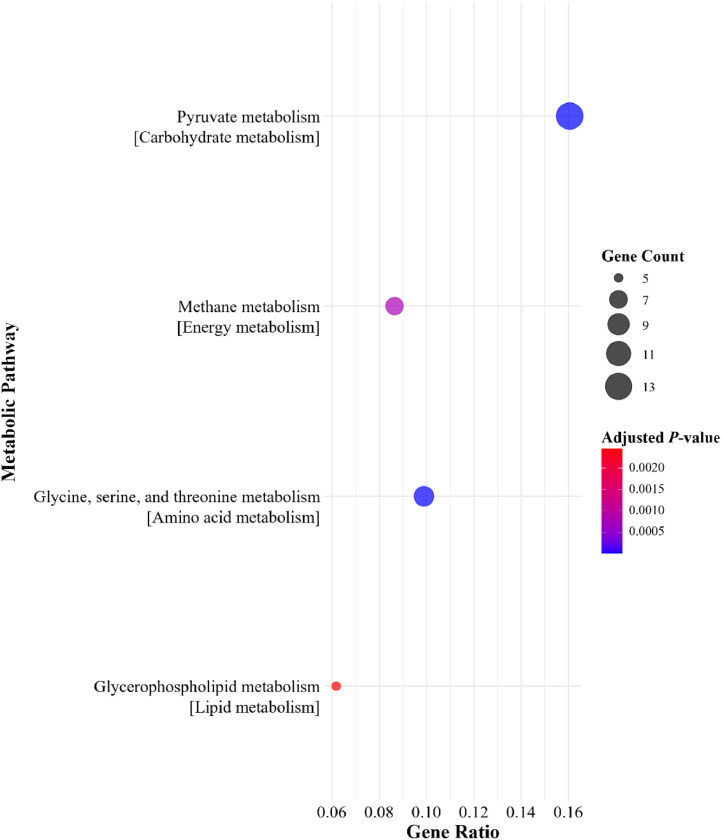
Downregulated metabolic pathways based on metagenome predicted from 16S rRNA gene abundances between YPM x Ross 708 male broilers provided a negative control (NC) with 500 FTU/kg of phytase^1^ and a NC (0 FTU/kg) diet on d 28. ^1^OptiPhos Plus (Huvepharma Inc., Peachtree City, GA).

**Fig. 3:**
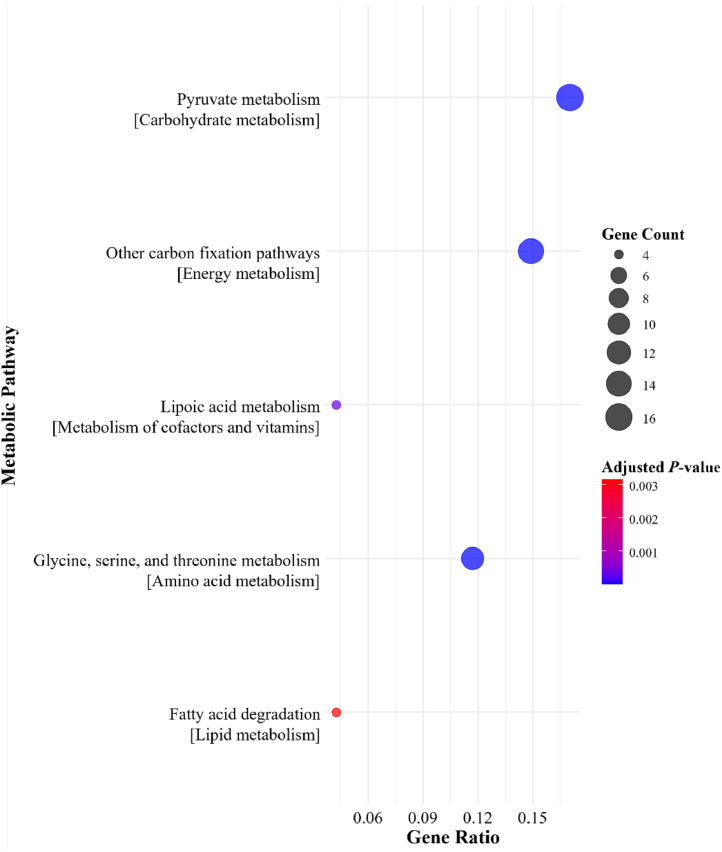
Upregulated metabolic pathways based on metagenome predicted from 16S rRNA gene abundances between YPM x Ross 708 male broilers provided a negative control (NC) with 1500 FTU/kg of phytase^1^ and a NC (0 FTU/kg) diet on d 28. ^1^OptiPhos Plus (Huvepharma Inc., Peachtree City, GA).

**Fig. 4:**
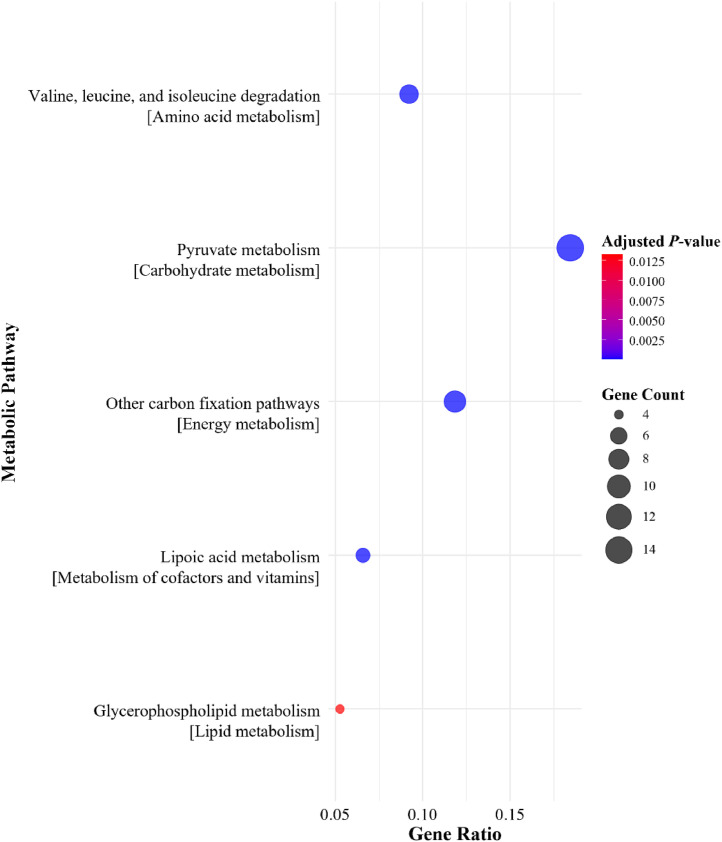
Downregulated metabolic pathways based on metagenome predicted from 16S rRNA gene abundances between YPM x Ross 708 male broilers provided a negative control (NC) with 1500 FTU/kg of phytase^1^ and a NC (0 FTU/kg) diet on d 28. ^1^OptiPhos Plus (Huvepharma Inc., Peachtree City, GA).

**Fig. 5:**
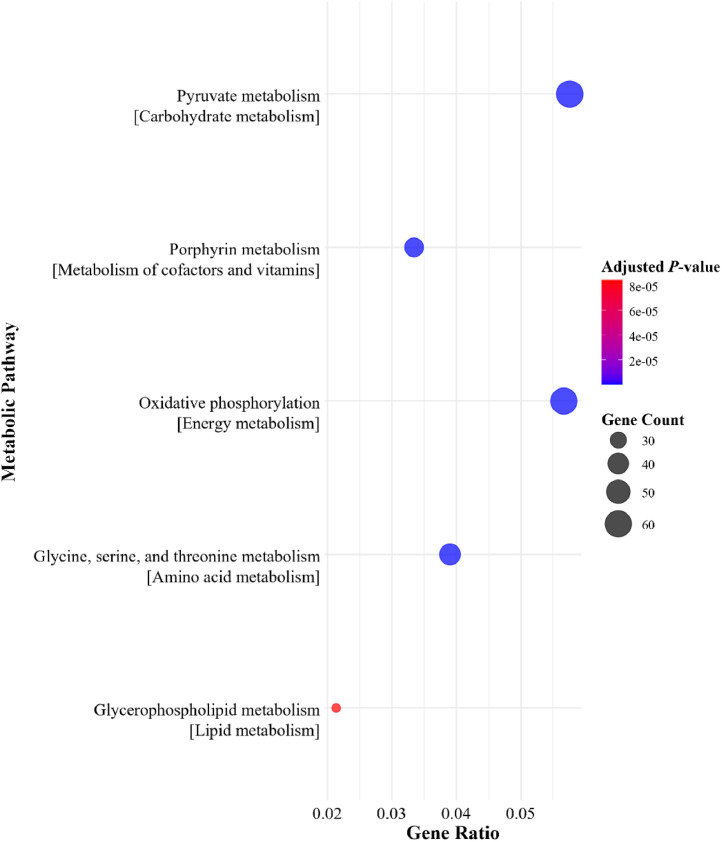
Downregulated metabolic pathways based on metagenome predicted from 16S rRNA gene abundances between YPM x Ross 708 male broilers provided a negative control (NC) with 500 FTU/kg of phytase^1^ and a NC (0 FTU/kg) diet on d 42. ^1^OptiPhos Plus (Huvepharma Inc., Peachtree City, GA).

Selected predicted bacterial metabolic pathways between the NC + phytase groups and the NC without phytase on d 28 and 42 are shown in Fig. 2, Fig. 3, Fig. 4, Fig. 5. No pathways primarily associated with phosphorus or calcium absorption or phytate degradation were regulated with 500 or 1500 FTU/kg of phytase. However, depending on sampling day, dietary phytase either upregulated or downregulated pathways involved in carbohydrate metabolism, metabolism of cofactors and vitamins, energy metabolism, amino acid metabolism, and lipid metabolism. In the cecal microbiome on d 28, dietary phytase at 500 FTU/kg downregulated metabolic pathways related to pyruvate metabolism, methane metabolism, Gly, Ser, and Thr metabolism, and glycerophospholipid metabolism (Fig. 2). Supplementing 1500 FTU/kg of phytase upregulated pathways related to pyruvate metabolism, other carbon fixation pathways, lipoic acid metabolism, Gly, Ser, and Thr metabolism, and fatty acid degradation (Fig. 3). Conversely, on d 28 using 1500 FTU/kg, downregulation was observed in pathways associated with Val, Leu, and Ile degradation, pyruvate metabolism, other carbon fixation pathways, lipoic acid metabolism, and glycerophospholipid metabolism (Fig. 4). As for the cecal microbiome on d 42, dietary phytase at 500 FTU/kg downregulated pyruvate metabolism, porphyrin metabolism, oxidative phosphorylation, Gly, Ser, and Thr metabolism, and glycerophospholipid metabolism (Fig. 5). Among these pathways, pyruvate metabolism, Gly, Ser, and Thr metabolism, lipoic acid metabolism, and glycerophospholipid metabolism were observed across multiple NC + phytase and NC comparisons. Detailed cecal microbiome metabolic pathway analyses comparing NC and NC + phytase inclusion are provided in Supplementary Data 3.

Pearson correlation coefficients between d 28 and 42 live performance measurements and intestinal health and physiology measurements are presented in Tables S1 and S2 (Supplementary Data 1). On d 28, most correlations occurred in the 500 FTU/kg group, including a weak positive correlation between VH and BW, moderate positive correlations between both VH:CD ratio and Pielou's evenness with BW, and a moderate negative correlation between Pielou's evenness and FCR (*P* ≤ 0.05). In the 1500 FTU/kg group, OF showed a moderate positive correlation with BW, while VH:CD ratio had a strong positive correlation with BW in the only BMD group (*P* ≤ 0.05). Pielou's evenness (only CB) and VH (both CB and BMD) had a moderate negative and positive correlation with FCR, respectively (*P* ≤ 0.05). In the group without MMFA, Pielou's evenness showed a strong negative correlation with AIDE (*P* ≤ 0.05). On d 42, VH showed a moderate positive correlation with CP digestibility (500 FTU/kg and only BMD) and AIDE (1500 FTU/kg), whereas a moderate negative correlation was observed with BW (500 FTU/kg) (*P* ≤ 0.05). In the 1500 FTU/kg and only BMD groups, VH:CD ratio had a weak and strong positive correlation with AIDE and CP digestibility, respectively (*P* ≤ 0.05). Pielou's evenness showed a strong negative (only CB) and moderate positive (both CB and BMD) correlation with FCR and AIDE, respectively (*P* ≤ 0.05). Observed features showed a moderate negative correlation with AIDE in the group without MMFA (*P* ≤ 0.05).

## Discussion

Phytase, CB, and BMD are frequently used as feed additives to improve the performance of broilers. Analysis of bird performance and nutrient digestibility indicated that BMD had a more positive impact compared to CB and revealed that broiler growth response to CB and BMD can be influenced by phytase concentration (Gulizia et al., 2024). As will be discussed later, intestinal health and physiology measurements responded more to phytase and CB than to BMD. Consequently, directly linking the present results to the live performance outcomes reported by Gulizia et al. (2024) is challenging. This is also supported by the Pearson correlation coefficients, which showed no consistent pattern of correlations between measurements on d 28 and 42. However, the 500 FTU/kg phytase group appeared more likely to exhibit correlations between these measurements. Thus, establishing a direct relationship between intestinal health and physiology measurements and live performance outcomes in response to these dietary interventions is complex. Nevertheless, the analyses of these data presented herein were undertaken to explore how phytase, CB, and BMD affect diversity and function of the intestinal microbiota, intestinal permeability, histomorphometry, and expression of tight junction genes.

Butyrate-based feed additives and BMD act mostly by modifying the intestinal microbiota (Bortoluzzi et al., 2017; Li et al., 2022), which then can change expression of tight junction genes, histomorphometry, and intestinal permeability. In addition, butyrate-based feed additives can have immunomodulatory effects (Zhou et al., 2014; Bortoluzzi et al., 2017). In contrast, phytase acts on the feed by degrading phytate, which releases phosphorus and reduces its anti-nutritional properties related to endogenous nitrogen losses (Cowieson et al., 2004, 2009). Reducing the intestinal flow of undigested protein and nitrogen can limit the substrate available for pathogenic bacteria (Qaisrani et al., 2015). Similarly, lowering endogenous nitrogen losses through phytase-mediated phytate degradation may also restrict the growth of pathogenic bacteria (Ptak et al., 2015; Moita et al., 2021). In this context, the effects of phytase, CB, and BMD on intestinal microbiota are of central importance. These effects have been studied before and yielded mostly similar results compared to the present study. Ultimately, the key results of this study are the interactions between phytase and the MMFA. In agreement with literature (Proctor and Phillips, 2019; Naghizadeh et al., 2022a), CB and BMD generally did not change alpha diversity of the cecal microbiota. However, interactions were observed with an alpha diversity response to the inclusion of MMFA in the 500 FTU/kg phytase group on d 28, while a response to MMFA occurred in the 1500 FTU/kg group on d 42. The inclusion of phytase or MMFA did not significantly change the composition of the cecal microbiota, as assessed by beta diversity. Lack of a significant effect from MMFA, i.e. feed additives intended to modulate the intestinal microbiota, on beta diversity was unexpected, but not unprecedented (Adhikari et al., 2023; Melaku et al., 2024). Alpha and beta diversity responses of the cecal microbiota to specific dietary feed additives can depend on the inclusion of additional feed additives, potentially explaining conflicting results in the literature.

Regulation of bacterial metabolic pathways is the most important aspect of the microbiome, potentially affecting host-microbiome interactions (Pan and Yu, 2014). There are limited papers which assessed the effects of phytase on predicted metabolic pathways in the intestinal microbiota of chickens. Depending on the concentration, phytase had a more pronounced effect on DEGs and predicted metabolic functions in the cecal microbiome compared to the MMFA. Complete dephosphorylation of phytate can liberate inositol (Kriseldi et al., 2021), which can further influence the intestinal microbiota (Yan et al., 2024). In the present study, phytase upregulated Gly, Ser, and Thr metabolism, which is the opposite result of Siegert et al. (2021) who reported that 1,500 FTU/kg of phytase decreased Gly, Ser, and Thr metabolism in the ileum microbiota. The reasons for conflicting results are unclear and may be related to the dietary inclusion of MMFA or the intestinal section sampled. In addition, the analytical tools used for assessing bacterial metabolic pathways limited the evaluation of interactions, hindering a clear understanding of how metabolic functions are regulated when both phytase and MMFA are supplemented. Siegert et al. (2021) also showed interactions between phytase and calcium levels, making a direct comparison difficult.

Exogenous phytase also changed pyruvate metabolism and lipoic acid metabolism within the cecal microbiome. Pyruvate metabolism is associated with energy production pathways (Turnbaugh et al., 2008), while lipoic acid is a necessary cofactor for reactions in pyruvate metabolism (Reed, 1957). Glycine, Ser, and Thr are glucogenic amino acids that have interconnected pathways with pyruvate, contributing to both amino acid and energy metabolism (Woolfson, 1983; He et al., 2021). Additionally, pyruvate metabolism has been positively correlated with *Alistipes* (Zhang et al., 2019), the most common genus observed on d 28 and 42 in the present study. All of this indicates that altered pyruvate metabolism is central to the observed changes in cecal microbiota function. Furthermore, glycerophospholipid metabolism was the only pathway consistently downregulated with phytase inclusion. Metabolites of glycerophospholipid metabolism have important roles in lipid metabolism, cellular membrane integrity, and signaling (Denich et al., 2003; Zhang and Rock, 2008). Upstream intestinal development, nutrient digestibility, and subsequent flow of substrates to the lower gastrointestinal tract may influence downstream microbial dynamics (Apajalahti et al., 2012; Quinger et al., 2024). The minimal effects of phytase and MMFA on the cecal microbiota suggest limited treatment impacts on intestinal permeability, histomorphometry, and tight junction gene expression. Further investigation is needed to understand the relationship between cecal microbiome dynamics and upper small intestinal physiology.

Intestinal permeability was not altered, and this lack of response to the addition of phytase or MMFA has been previously observed (Zanu et al., 2020a; Naghizadeh et al., 2022b). Another method to assess intestinal health is through the morphological development of the gastrointestinal tract, where higher VH and lower CD indicate better intestinal health and improved nutrient absorption capacity (Ducatelle et al., 2018; Bindari and Gerber, 2022). Some available literature suggests that phytase (Moita et al., 2021) and butyrate-based feed additives (Ahsan et al., 2016) can enhance intestinal VH and VH:CD ratio. Previous research also indicates that bacitracin alone did not improve intestinal morphology (Letlole et al., 2021; Adhikari et al., 2023). These findings are only in partial agreement with the results of the present study. While CB increased d 42 jejunal VH compared to BMD, phytase affected jejunal histomorphometry only on d 42 in birds not supplemented with MMFA. As with the cecal microbiota, one possible cause of these partial discrepancies may be interactions between dietary feed additives, some of which were also observed in the present study. Combining 1500 FTU/kg of phytase with both CB and BMD decreased d 28 VH compared to 500 FTU/kg. Similarly, higher phytase concentrations combined with sodium butyrate (1500 vs. 750 FTU/kg; Layter et al., 2021) increased CD, while higher phytase concentrations combined with both salinomycin and zinc bacitracin (1500 vs. 500 FTU/kg; Zanu et al., 2020b) reduced VH. Given the limited literature, there is possibly an extent to which higher phytase concentrations combined with butyrate or antibiotics can improve intestinal histomorphometry, though the specific mechanism remains unclear.

The final set of measurements evaluated were the expression of tight junction and mucin producing genes, which play critical roles in maintaining intestinal integrity and function by regulating the paracellular movement of ions (González-Mariscal et al., 2003; Awad et al., 2017). Claudins 1, 4, and 5 are barrier-forming tight junction proteins that can prevent paracellular movement of cations, pathogens, and feed contaminants in the gastrointestinal tract, while CL-2 has a pore-forming role that can facilitate the movement of cations across the epithelium (Günzel and Yu, 2013; Awad et al., 2017). Zonula occludens support the structure and function of claudins (González-Mariscal et al., 2003; Lee, 2015), with ZO-1 and ZO-2 having independent effects on claudins during tight junction formation (Umeda et al., 2006). Although not a tight junction gene, MUC-2 is crucial for mucin production, which serves as the first line of defense against pathogens in the gastrointestinal tract (Jiang et al., 2013; Duangnumsawang et al., 2021). Even more than the previously discussed factors, reports on the influence of phytase and MMFA on intestinal tight junction and mucin gene expression are conflicting. These effects vary depending on dosing level, other dietary factors (Zanu et al., 2020a; Luo et al., 2021), and disease state (Song et al., 2017; Shi et al., 2024). The complexity is evident in the present findings, where interactions were observed in the expression of CL-1, CL-4, CL-5, and ZO-2 on d 28 as well as CL-2 on d 42. Interestingly, less interactions were observed on d 42, suggesting that the effect of phytase and MMFA becomes more uniform in older birds with a more mature intestinal microbiota. While it remains unclear whether the up- or down-regulation of these genes benefits intestinal health, and no optimal level has been established, the regulation of tight junction gene expression appears to have supported intestinal integrity, as no differences were observed in intestinal permeability. Furthermore, expression of tight junction and mucin genes were altered but this does not always refer to protein translation (Greenbaum et al., 2003).

In the present study, more interaction effects on jejunal histomorphometry, jejunal gene expression, and cecal alpha diversity were observed on d 28 compared to d 42 (7 vs. 3 significant interactions). Possibly, the effects of phytase and MMFA were influenced by bird age and gastrointestinal tract physiology. Discrepancies between sampling days could be attributed to shifts in microbial populations (Lu et al., 2003), age-related changes in intestinal tight junction protein expression (von Buchholz et al., 2021; Abascal-Ponciano et al., 2022), and alterations in diet composition (Marmion et al., 2021). Although the jejunal microbiome was not assessed, a distinct shift in cecal microbiome beta diversity between sampling days (Supplementary Data 1, Fig. S4) suggests that microbial communities may have also changed over time. This shift over time is supported by the log fold change in genera (Supplementary Data 1, Fig. S1) as dietary treatments had less influence on d 42 as compared to d 28. As broilers age, their intestinal development (Ravindran and Abdollahi, 2021) and microbial populations (Lumpkins et al., 2010; Rama et al., 2023) tend to stabilize, which may explain the reduced sensitivity to dietary interventions observed on d 42 in this study. However, the specific timeframe for microbial stabilization in broilers remains ambiguous (Feye et al., 2020).

Dietary supplementation of phytase (500 or 1,500 FTU/kg), CB (0.5 g supplement/kg of diet), and BMD (55 ppm) influenced indicators of intestinal health and physiology of broilers. While intestinal permeability remained unaffected and changes in jejunal histomorphometry and cecal microbiome diversity were minimal, phytase, MMFA, and, at times, their interaction, significantly affected jejunal tight junction and mucin gene expression and predicted bacterial metabolic function. In conclusion, these findings highlight the complex interactions between feed additives, particularly the dependence of MMFA effects on phytase concentration. Given that phytase supplementation in broiler diets is commonplace, understanding possible synergistic or antagonistic relationships between phytase, CB, and BMD is essential for improving intestinal health and performance. Results obtained when investigating effects of MMFA with a certain inclusion of phytase cannot reliably be extrapolated to other inclusion concentrations.

## Funding source

This research was funded by a USDA-ARS Cooperative Agreement (G00014614 - Dietary manipulations to improve gut health in broiler production).

## Declaration of competing interest

The authors declare that they have no known competing financial interests or personal relationships that could have appeared to influence the work reported in this paper.

## Appendix Supplementary materials

Image, application 1

Image, application 2

Image, application 3

## Footnotes

Supplementary material associated with this article can be found, in the online version, at doi:10.1016/j.psj.2025.104862.

## Footnotes

1 The accession includes additional samples that were excluded from the cecal microbiome analyses, as only 1 bird per pen was analyzed.
