# Supplementary material for: Response of YPM x Ross 708 male broilers to diets containing varying inclusions of phytase, calcium butyrate, and bacitracin methylene disalicylate during the grower and finisher periods–part 2: Intestinal health and physiology
Source: Poult Sci. 2025 Jan 31;104(3):104862. doi: 10.1016/j.psj.2025.104862 (PMC12011101; doi:10.1016/j.psj.2025.104862)
Supplement: Supplementary file 1 [file mmc1.docx]

**Response of YPM x Ross 708 male broilers to diets containing varying inclusions of phytase, calcium butyrate, and bacitracin methylene disalicylate during the grower and finisher periods–part 2: intestinal health and physiology**

Joseph P. Gulizia, Zubair Khalid, Maria T. Terra-Long, Jose I. Vargas, Jose R. Hernandez, Wilmer J. Pacheco, James Krehling, Ken Macklin, William A. Dozier, III, Klint W. McCafferty, and Ruediger Hauck

**SUPPLEMENTARY DATA 1**

Supplementary data on cecal microbiome composition, beta diversity, and correlations with performance and intestinal health in YPM x Ross 708 male broilers.


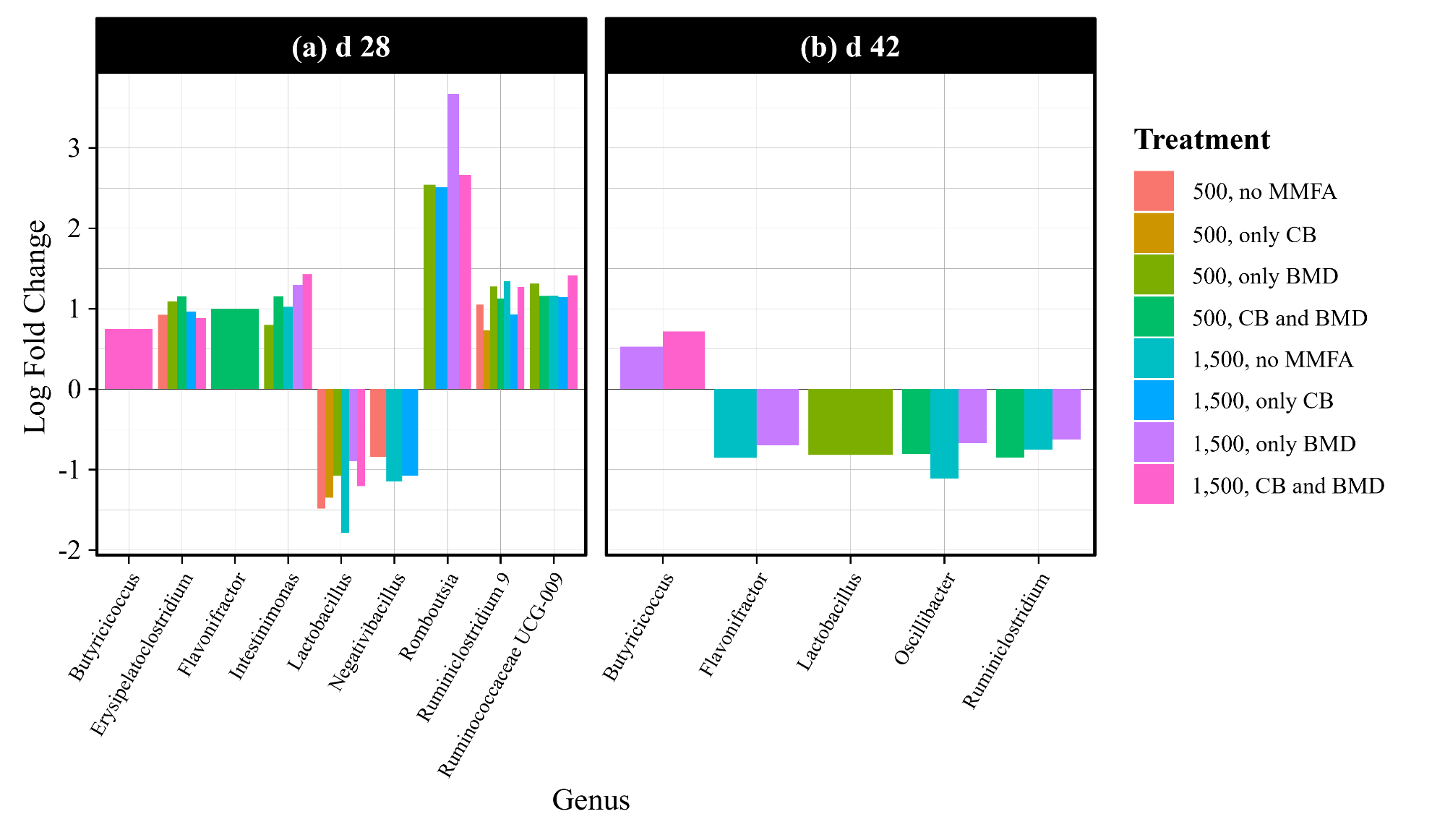


| **Figure S1**. Differential abundance indicating log fold change in bacterial genera for (**a**) d 28 and (**b**) d 42 cecal microbiome of YPM x Ross 708 male broilers provided a negative control (NC) diet varying in phytase (500 or 1,500 FTU/kg) and microbiota modulating feed additive inclusion (MMFA; none, only calcium butyrate (CB), only bacitracin methylene disalicylate (BMD), or both CB and BMD). All log fold changes are significant (*P* ≤ 0.05). |
| --- |


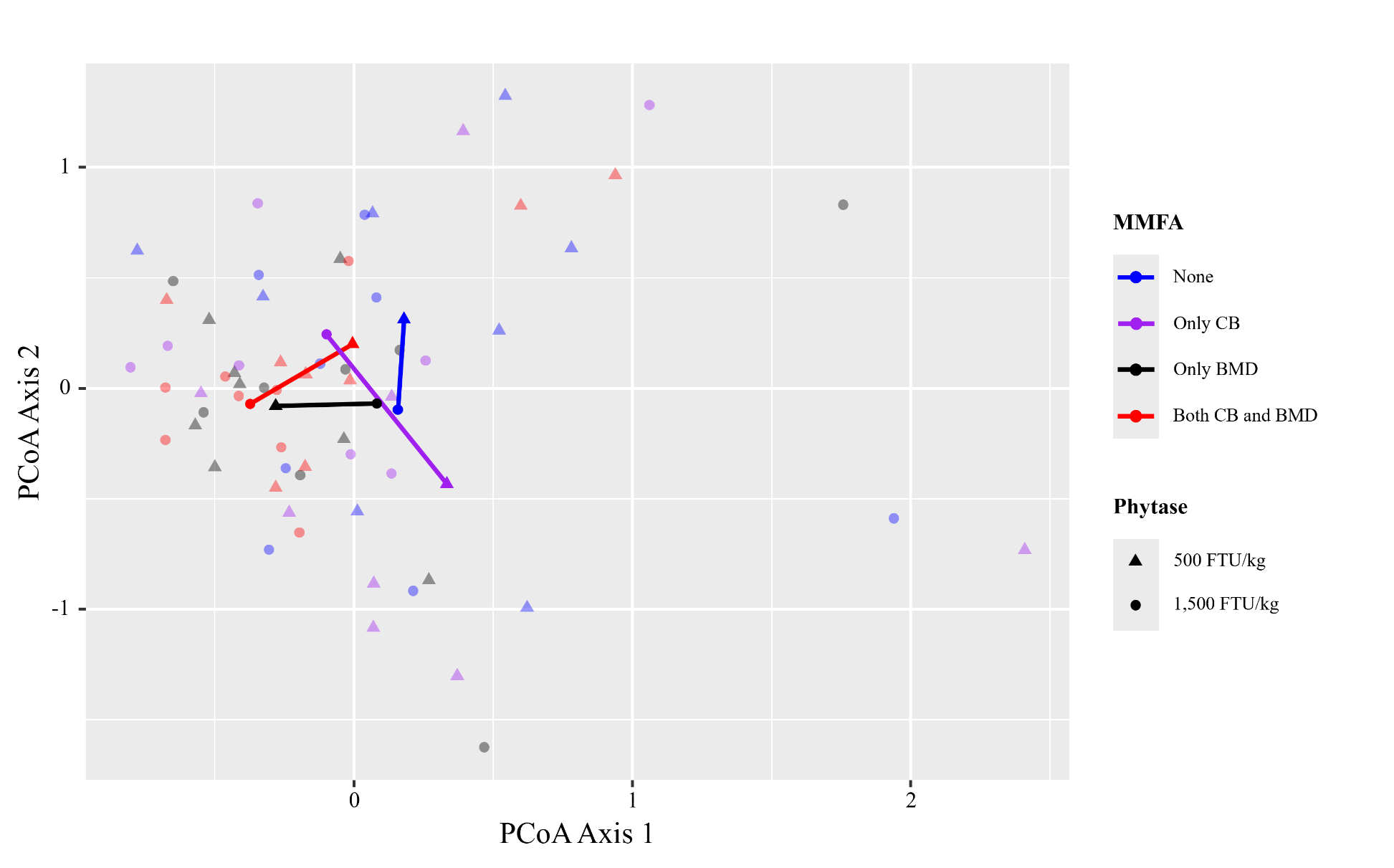


| **Figure S2**. Beta diversity based on unweighted UniFrac distances for d 28 cecal microbiome of YPM x Ross 708 male broilers provided a negative control diet varying in phytase, calcium butyrate (CB), and bacitracin methylene disalicylate (BMD) inclusion. Interactions between phytase and microbiota modulating feed additives (MMFA) are plotted using mean centroids of the respective groups (*P* = 0.175). |
| --- |


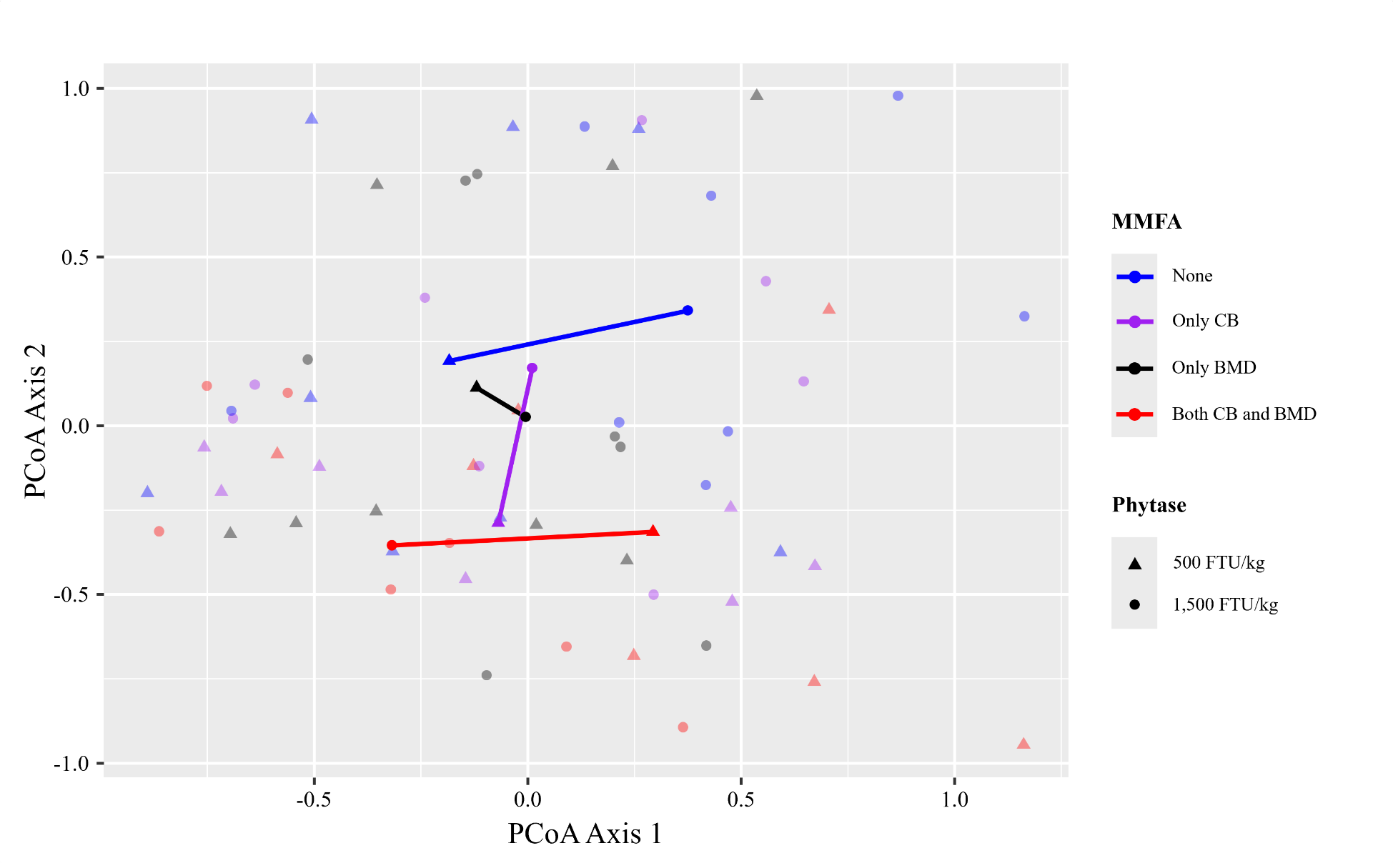


| **Figure S3**. Beta diversity based on unweighted UniFrac distances for d 42 cecal microbiome of YPM x Ross 708 male broilers provided a negative control diet varying in phytase, calcium butyrate (CB), and bacitracin methylene disalicylate (BMD) inclusion. Interactions between phytase and microbiota modulating feed additives (MMFA) are plotted using mean centroids of the respective groups (*P* = 0.070). |
| --- |

| **Table S1**. Pearson correlation coefficients (r) between d 28 live performance measurements and intestinal health and physiology measurements of YPM x Ross 708 male broilers provided a negative control diet varying in phytase, calcium butyrate (CB), and bacitracin methylene disalicylate (BMD) inclusion. | | | | |
| --- | --- | --- | --- | --- |
| Measurement | BW | Feed conversion ratio | CP digestibility | AIDE^1^ |
| Phytase, FTU/kg, main effect |  |  |  |  |
| 500 |  |  |  |  |
| Villus height | 0.3630* | 0.1350 | 0.1339 | -0.0067 |
| VH:CD ratio | 0.5133** | -0.2663 | 0.1419 | 0.2029 |
| Pielou's evenness | 0.4449* | -0.5397** | 0.2271 | -0.1560 |
| Observed features | -0.0770 | -0.1492 | -0.0158 | -0.0801 |
| 1,500 |  |  |  |  |
| Villus height | 0.0225 | 0.1919 | 0.0048 | 0.0023 |
| VH:CD ratio | -0.0088 | 0.2192 | 0.3500 | 0.2626 |
| Pielou's evenness | 0.0661 | 0.0652 | -0.0543 | -0.0981 |
| Observed features | 0.4422* | -0.2941 | 0.1638 | 0.0473 |
| MMFA^2^, main effect |  |  |  |  |
| None |  |  |  |  |
| Villus height | 0.2819 | 0.1739 | 0.1736 | -0.1935 |
| VH:CD ratio | -0.0093 | 0.2625 | 0.0076 | 0.1225 |
| Pielou's evenness | -0.2449 | 0.4308 | -0.4451 | -0.5974* |
| Observed features | -0.0031 | 0.0839 | -0.0570 | -0.0182 |
| Only CB |  |  |  |  |
| Villus height | 0.1708 | 0.4090 | -0.1696 | 0.1272 |
| VH:CD ratio | -0.3125 | -0.1826 | -0.0311 | 0.1808 |
| Pielou's evenness | 0.1855 | -0.5816* | 0.0649 | 0.1884 |
| Observed features | 0.0113 | -0.5288 | -0.1212 | -0.0882 |
| Only BMD |  |  |  |  |
| Villus height | 0.4201 | -0.4589 | 0.0704 | 0.1747 |
| VH:CD ratio | 0.7012** | -0.1589 | 0.3189 | 0.4541 |
| Pielou's evenness | 0.0077 | -0.3089 | -0.0019 | -0.4608 |
| Observed features | -0.3743 | -0.3255 | -0.3979 | -0.0027 |
| Both CB and BMD |  |  |  |  |
| Villus height | -0.1956 | 0.5398* | -0.2394 | -0.2604 |
| VH:CD ratio | -0.1602 | 0.4750 | -0.0673 | 0.0267 |
| Pielou's evenness | 0.0739 | 0.1519 | 0.2555 | 0.0852 |
| Observed features | 0.1410 | -0.0058 | -0.0653 | -0.0168 |
| **P* ≤ 0.05, ***P* ≤ 0.01, ****P* ≤ 0.001  ^1^AIDE = apparent ileal digestible energy.  ^2^MMFA = microbiota modulating feed additives. | | | | |

| **Table S2**. Pearson correlation coefficients (r) between d 42 live performance measurements and intestinal health and physiology measurements of YPM x Ross 708 male broilers provided a negative control diet varying in phytase, calcium butyrate (CB), and bacitracin methylene disalicylate (BMD) inclusion. | | | | |
| --- | --- | --- | --- | --- |
| Measurement | BW | Feed conversion ratio | CP digestibility | AIDE^1^ |
| Phytase, FTU/kg, main effect |  |  |  |  |
| 500 |  |  |  |  |
| Villus height | -0.4234* | 0.2672 | 0.4682** | 0.1072 |
| VH:CD ratio | -0.1489 | 0.2344 | 0.1300 | 0.0508 |
| Pielou's evenness | 0.1107 | -0.0700 | 0.1218 | 0.1195 |
| Observed features | -0.2596 | 0.1190 | -0.0070 | -0.0902 |
| 1,500 |  |  |  |  |
| Villus height | -0.1721 | 0.0424 | 0.2851 | 0.4017* |
| VH:CD ratio | -0.0245 | 0.0191 | 0.3022 | 0.3557* |
| Pielou's evenness | 0.3511 | -0.2892 | -0.1949 | -0.0565 |
| Observed features | 0.2123 | 0.0126 | -0.2820 | -0.1654 |
| MMFA^2^, main effect |  |  |  |  |
| None |  |  |  |  |
| Villus height | -0.0089 | 0.0472 | 0.5016 | 0.2850 |
| VH:CD ratio | 0.2231 | 0.3323 | 0.1557 | 0.1230 |
| Pielou's evenness | 0.4358 | -0.3010 | 0.0563 | -0.3553 |
| Observed features | 0.4207 | -0.3879 | -0.2993 | -0.5348* |
| Only CB |  |  |  |  |
| Villus height | 0.2146 | -0.0644 | 0.2286 | 0.2603 |
| VH:CD ratio | 0.0808 | -0.1122 | -0.1977 | 0.4034 |
| Pielou's evenness | 0.5216 | -0.6112* | 0.2693 | -0.1042 |
| Observed features | -0.0406 | 0.0927 | 0.2299 | 0.2348 |
| Only BMD |  |  |  |  |
| Villus height | -0.4719 | 0.4483 | 0.5167* | 0.1840 |
| VH:CD ratio | -0.4054 | 0.3059 | 0.7139** | 0.2624 |
| Pielou's evenness | 0.2049 | 0.1313 | -0.2212 | 0.0995 |
| Observed features | -0.3182 | 0.4123 | -0.1426 | 0.0247 |
| Both CB and BMD |  |  |  |  |
| Villus height | 0.0513 | 0.0621 | 0.1525 | 0.1956 |
| VH:CD ratio | 0.1802 | -0.1416 | 0.1064 | -0.0445 |
| Pielou's evenness | -0.2057 | -0.2506 | -0.0593 | 0.5879* |
| Observed features | 0.0585 | -0.1281 | -0.3764 | 0.0719 |
| **P* ≤ 0.05, ***P* ≤ 0.01, ****P* ≤ 0.001  ^1^AIDE = apparent ileal digestible energy.  ^2^MMFA = microbiota modulating feed additives. | | | | |


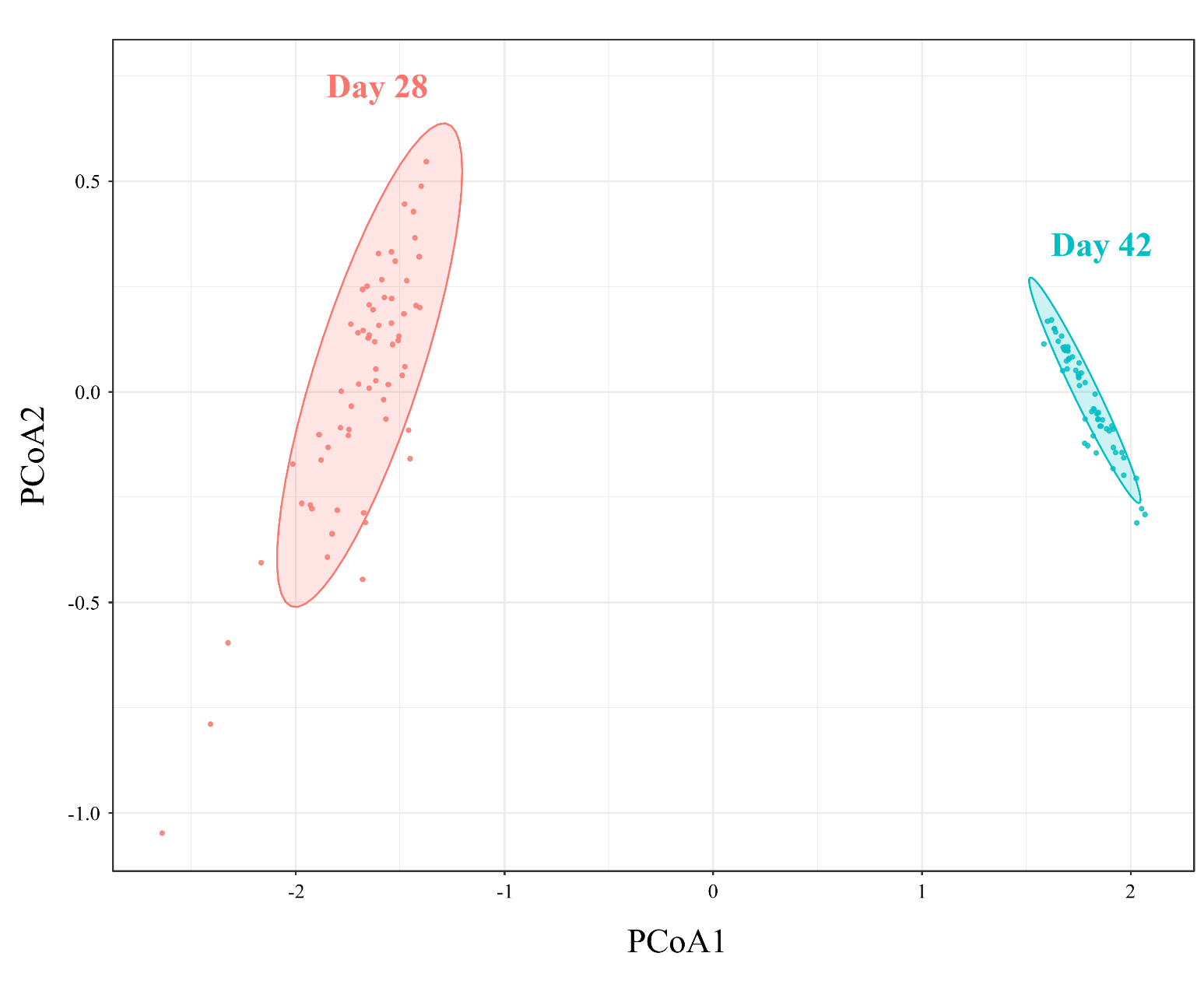


| **Figure S4**. Beta diversity based on unweighted UniFrac distances for d 28 and 42 cecal microbiome of YPM x Ross 708 male broilers provided a negative control diet varying in phytase, calcium butyrate, and bacitracin methylene disalicylate inclusion. Beta diversity between sampling days was significant (*P* = 0.001). |
| --- |
